# Enteric Duplication Cyst Located at the Posterior Tongue: A Rare Case Report and Review of the Literature

**DOI:** 10.1155/2015/951878

**Published:** 2015-02-23

**Authors:** Bircan Savran, Cuneyt Kucur, Cengiz Kocak, Isa Ozbay, Mehmet Huseyin Metineren, Yasin Tugrul Karakus

**Affiliations:** ^1^Department of Pediatric Surgery, Faculty of Medicine, Dumlupinar University, 43000 Kutahya, Turkey; ^2^Department of Otolaryngology, Faculty of Medicine, Dumlupinar University, 43000 Kutahya, Turkey; ^3^Department of Pathology, Faculty of Medicine, Dumlupinar University, 43000 Kutahya, Turkey; ^4^Department of Pediatrics, Faculty of Medicine, Dumlupinar University, 43000 Kutahya, Turkey

## Abstract

The lingual localization of an enteric duplication is extremely rare but may present with respiratory and feeding problems that require emergency intervention. A 7-month-old boy was brought to our clinic with feeding difficulties and tongue swelling. Physical examination showed a cystic lesion located near the left side of the tongue base that caused tongue protrusion to the contralateral side. During surgery, a 3-cm diameter opaque thick-walled cyst was found to be very closely adherent to the base of tongue, which was excised in its entirety. Following surgery, the patient fed during the early postoperative period and no complications were observed other than hypersalivation. On histological examination, a cystic lesion lined with intestinal mucosa and goblet cells was found. We present the rare case of a duplication cyst of the posterior tongue, with a literature review.

## 1. Introduction

Enteric duplication describes heterotopy of the gastrointestinal mucosa, which is a rare congenital malformation that may vary greatly in presentation, size, location, and symptoms. Duplication cysts can occur anywhere in the gastrointestinal tracts from the mouth to the anus [1]. The size of the duplication determines the timing of presentation. Larger sizes cause more acute admissions with dyspnea or dysphagia. Enteric duplications are generally symptomatic within the first year of life with consequent episodes of intestinal obstruction or palpable masses. Lingual localization is extremely rare and may present with respiratory and feeding problems that require emergency intervention [2]. We present a case of a duplication cyst of the posterior tongue, with a literature review.

## 2. Case Report

**Figure 1:**
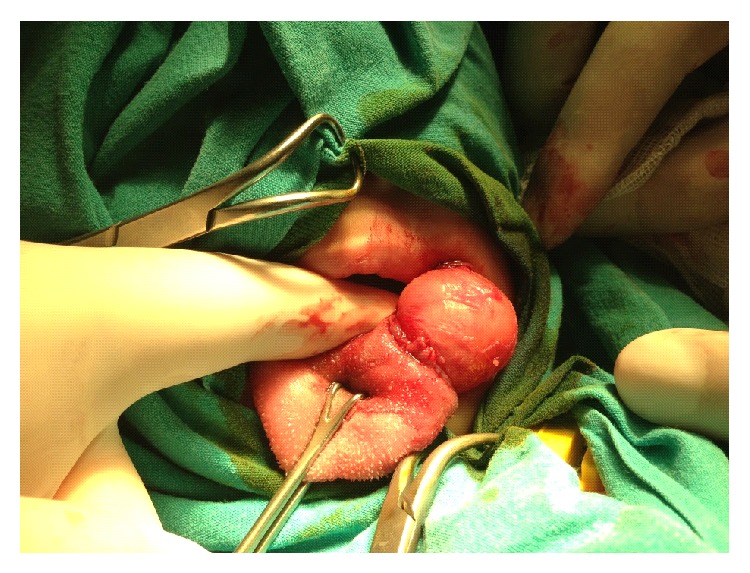
During surgery, a 3-cm diameter cystic lesion was seen intraoperatively.

**Figure 2:**
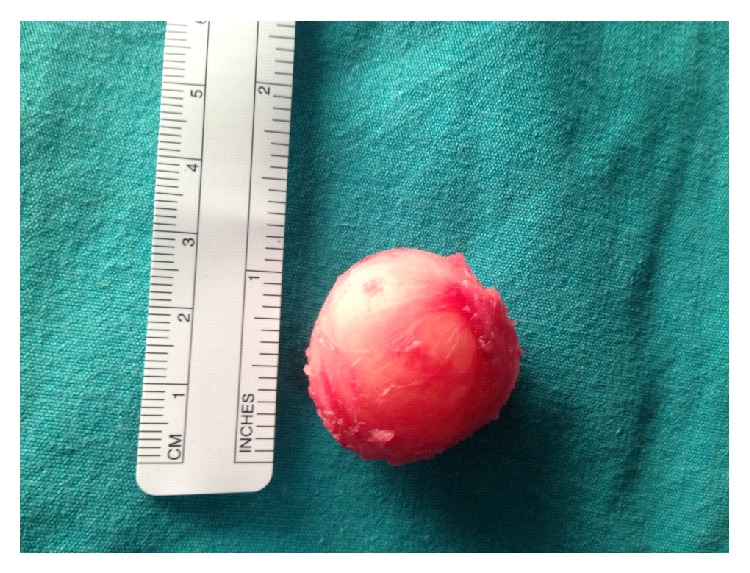
3-cm diameter cystic lesion was completely excised intact from the tongue base.

A 7-month-old boy was brought to our clinic with feeding difficulties and swelling of the tongue. On physical examination; we noticed a cystic lesion located near the left side of the tongue base which was causing protrusion of the tongue to the contralateral side. The child did not have any other anomalies on physical examination and his medical history was otherwise unremarkable. The patient was admitted for surgery, during which a 3-cm diameter opaque thick-walled cyst was identified to be very closely adherent to the base of the tongue. The total excision of the cyst via bipolar diathermy was performed under general anesthesia (Figures 1 and 2). We made preparation for emergent tracheostomy before the surgery in case orotracheal intubation is not possible; however no respiratory complications occurred during intubation or extubation.

**Figure 3:**
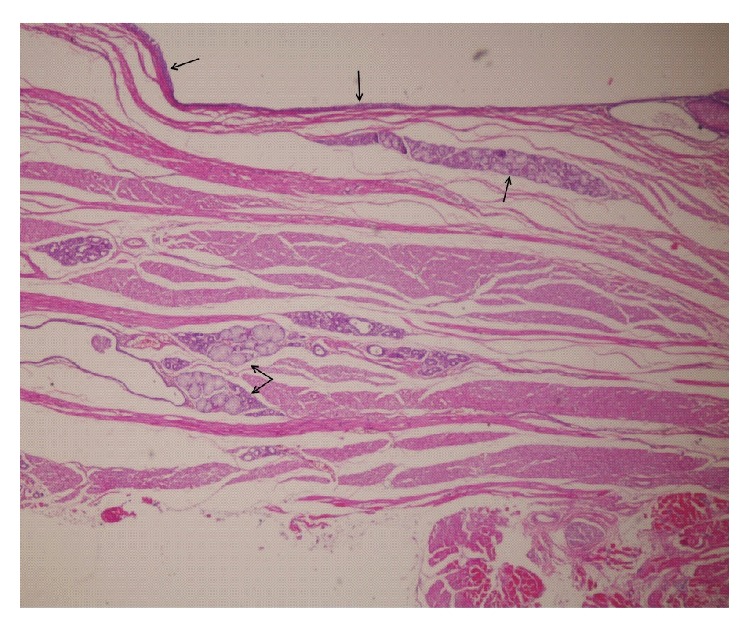
Histological section showing intestinal type columnar epithelium lining the cyst wall (hematoxylin and eosin; original magnification, ×40).

**Figure 4:**
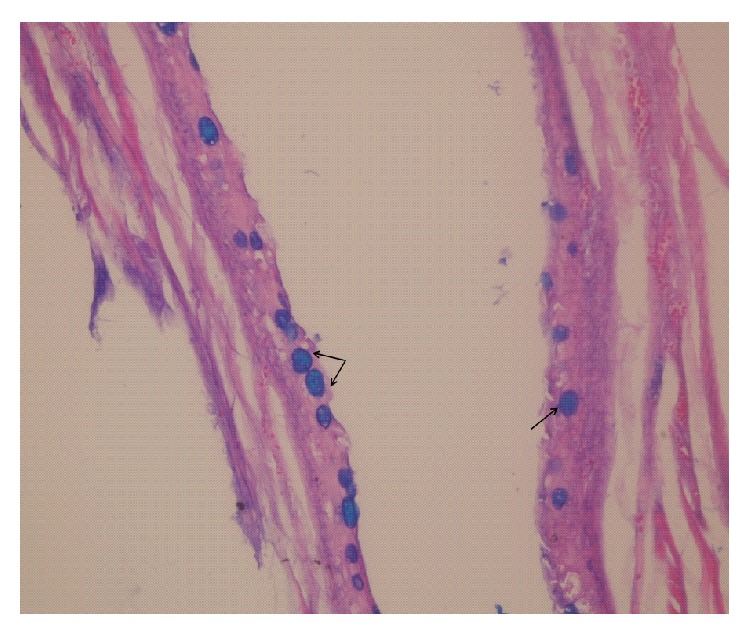
Histological section showing goblet cells located in the intestinal epithelium (Alcian blue; original magnification, ×100).

Following surgery, the patient fed during the early postoperative period and no complications were observed apart from hypersalivation. For histopathological examination, the excised specimen was fixed in 10% formalin, embedded in paraffin, sectioned into 4-*μ*m thick sections, and stained with hematoxylin and eosin (H&E) and Alcian blue, before being examined under light microscopy (Olympus BX51, Tokyo, Japan). The gross specimen measured 3 × 2.5 × 2.5 cm and consisted of a unilocular cystic mass that contained mucoid material. Microscopic examination indicated that the cystic lesion was lined with intestinal mucosa accompanied by goblet cells. Stratified squamous epithelium was also present in some areas (Figures 3 and 4).

## 3. Discussion

Oral cysts are rarely lined entirely by gastrointestinal epithelium. Heterotopic oral gastrointestinal cysts (e.g., enterocystomas, enteric duplication cysts) are usually considered to be choristomas or histologically normal tissue that is located in an abnormal position [3]. The etiology of such cysts is undetermined, but the generally accepted theory is that the lesion originates from islands of endodermal cells that prevent the fusion of the lateral lingual protuberances and the tuberculum impar during the third week of embryonic development [4, 5]. Although enteric duplication cysts can be found anywhere from the mouth to the anus, enteric duplication cysts of the tongue are unusual and can be confused with dermoid cysts, hemangiomas, lingual thyroid remnants, ranulas, and cystic hygromas. These enteric duplication cysts are either embedded deeply within the tongue or present as superficial, movable nodules of the lingual dorsum or oral floor [6]. Enteric duplications were observed in the anterior part of tongue in 60% of reported cases and are more common in boys [7–9].

During the early postnatal period, respiratory and feeding difficulties may be observed depending on the location and size of the cyst. Larger cysts present with dyspnea or dysphagia and symptoms are generally more acute. Although fine-needle aspiration of the cyst content can give temporary relief during the neonatal period, definitive treatment can only be achieved by total excision of the cyst via laser ablation or bipolar diathermy [1, 10, 11].

Although enteric duplication cysts can be lined by gastric, intestinal, colonic, or respiratory epithelium, the majority consist of gastric-type epithelium. Pathologically, cysts range from having only a mucosal lining, to having a full-thickness duplication that consists of the mucosa, submucosa, and muscularis propria [12, 13]. In the case presented herein, the cyst consisted of only an intestinal mucosal lining and the submucosa or muscularis propria were not present. Mueller and Callanan [4] and Satish Kumar et al. [13] reported two cases of enteric duplication cysts that were lined with heterotrophic gastric mucosa. Lingual duplication cysts associated with a combination of gastrointestinal and respiratory epithelium have been described by several previous reports [14–16]. The presence of colonic mucosa was reported by Awouters and Reychler [5], and Azañero et al. [17] reported two lingual cysts that were lined solely with respiratory epithelium. Grime reported a giant enterocystoma within the tongue and submandibular triangle that contained heterotopic gastric and intestinal epithelium [18]. Manor et al. [19] reviewed 53 lingual cysts that were reported in the literature between 1942 and 1997 and added a single case of their own. According to their study, 12 of the 52 reported cases were lined by respiratory epithelium, 25 were lined by gastrointestinal epithelium, and 15 were mixed. In the literature, very few cases of duplication cysts located within the area of the posterior tongue have been reported. Herein, we presented a case of an unusual location of a duplication cyst that contained heterotopic intestinal mucosa.

## 4. Conclusion

Duplication cysts should be considered as part of the differential diagnosis of a lingual cystic mass in an infant. The early surgical excision of such lesions in symptomatic patients is indicated to avoid failure-to-thrive and respiratory distress.

## References

[1] Reddy E. V., Mohan G. R., Prasad G. R., Sathyanarayana G. (2011). Lingual gastric duplication cyst in a new born. *Indian Journal of Otolaryngology and Head and Neck Surgery*.

[2] Blanchard M., Kadlub N., Boudjemaa S. (2012). Tongue cyst in children: foregut duplication, a possible diagnosis. *Revue de Stomatologie et de Chirurgie Maxillo-Faciale*.

[3] Lee C. M. A., Damm D., Neville B. W., Allen C., Bouquot J. (2008). *Oral and Maxillofacial Pathology*.

[4] Mueller D. T., Callanan V. P. (2007). Congenital malformations of the oral cavity. *Otolaryngologic Clinics of North America*.

[5] Awouters P., Reychler H. (1991). Enteric duplication in the oral cavity. *International Journal of Oral and Maxillofacial Surgery*.

[6] Lipsett J., Sparnon A. L., Byard R. W. (1993). Embryogenesis of enterocystomas-enteric duplication cysts of the tongue. *Oral Surgery Oral Medicine and Oral Pathology*.

[7] El-Bitar M. A., Milmoe G., Kumar S. (2003). Intralingual foregut duplication, cyst in a newborn. *Ear, Nose and Throat Journal*.

[8] Eaton D., Billings K., Timmons C., Booth T., Biavati J. M. J. (2001). Congenital foregut duplication cysts of the anterior tongue. *Archives of Otolaryngology—Head and Neck Surgery*.

[9] Willner A., Feghali J., Bassila M. (1991). An enteric duplication cyst occurring in the anterior two-thirds of the tongue. *International Journal of Pediatric Otorhinolaryngology*.

[10] Hambarde S., Bendre P., Taide D. (2011). Foregut duplication cyst presenting as lingual swelling: case report and review of literature. *National Journal of Maxillofacial Surgery*.

[11] Madan H. K., Swain L., Borkar J. (2012). Anesthetic management of a neonatal lingual gastric duplication cyst: report of a rare case. *Journal of Anesthesia*.

[12] Tucker R., Maddalozzo J., Chou P. (2000). Sublingual enteric duplication cyst. *Archives of Pathology and Laboratory Medicine*.

[13] Satish Kumar K., Joshi M., Vishwanath N., Akhtar T., Oak S. (2006). Neonatal lingual gastric duplication cyst: a rare case report. *Journal of Indian Association of Pediatric Surgeons*.

[14] Mirchandani R., Sciubba J., Gloster E. S. (1989). Congenital oral cyst with heterotopic gastrointestinal and respiratory mucosa. *Archives of Pathology and Laboratory Medicine*.

[15] Ameh E. A., Mshelbwala P. (2002). Intralingual foregut duplication cyst: a case report. *The Nigerian Postgraduate Medical Journal*.

[16] Ohbayashi Y., Miyake M., Nagahata S. (1997). Gastrointestinal cyst of the tongue: a possible duplication cyst of foregut origin. *Journal of Oral and Maxillofacial Surgery*.

[17] Azañero W. D., Mazzonetto R., León J. E., Vargas P. A., Lopes M. A., de Almeida O. P. (2009). Lingual cyst with respiratory epithelium: a histopathological and immunohistochemical analysis of two cases. *International Journal of Oral and Maxillofacial Surgery*.

[18] Grime P. D. (1990). Giant enterocystoma within an infant's tongue. *Journal of Laryngology and Otology*.

[19] Manor Y., Buchner A., Peleg M., Taicher S. (1999). Lingual cyst with respiratory epithelium: an entity of debatable histogenesis. *Journal of Oral and Maxillofacial Surgery*.

